# Complementary and alternative medicine (CAM) in pediatric rheumatology: an European perspective

**DOI:** 10.1186/1546-0096-9-S1-P238

**Published:** 2011-09-14

**Authors:** G Fadanelli, F Vittadello, G Martini, ME Zannin, G Zanon, F Zulian

**Affiliations:** 1 Rheumatology Unit, Dept. of Pediatrics, University of Padua, Padua, Italy

## Background

To analyse the use of complementary and alternative medicine (CAM) in children with rheumatic diseases, followed at a Pediatric Rheumatology Centre in Italy.

## Methods

Parents of children with different kind of chronic rheumatic diseases anonymously completed a questionnaire about past or current use of CAM. Two groups of patients were considered: group A consisted in children who were still attending the centre; group B consisted in children who did not attend the clinic for more than one year.

## Results

150 completed surveys were analyzed: 22 paediatric patients (14,7%), 10/100 in group A and 12/50 in group B, used CAM to treat their diseases. The therapies more used were homeopathy, herbal remedies, vitamins and minerals. We observed a significantly greater use of CAM among patients who did not attend the clinic for more than one year (24%) as compared to those who were regularly checked (10%) (p=0,022). Parents’ use of CAM was significantly related with its use for their children (p=0,001). A bad outcome, probably related to the exclusive use of alternative treatments, was observed in three out of six patients who had completely stopped the traditional immunosuppressive drugs.

## Conclusions

Physicians should be aware of the use of CAM particularly in patients who skip the regular check visits. The use of CAM to treat childhood rheumatic conditions in Italy seems to be lower than in North America.

